# Autonomic Nervous System Responses to Rest and Exertion Following Concussion (the Measuring ANSWERS Study): Protocol for a 2-Aim Cross-Sectional and Longitudinal Observational Study

**DOI:** 10.2196/103400

**Published:** 2026-08-14

**Authors:** Wesley R Cole, Aidan E Finegan, Adam W Kiefer, Aaron M Sinnott, Meredith Mann, Baiming Zou, Meisheng Xiao, Brittany L Heikke, Dominic Willoughby, Ryan MacPherson, Katelyn A Holter-Soerensen, Chloe D Campbell, Madison C Chandler, Johna K Register-Mihalik, Jason P Mihalik

**Affiliations:** 1 Department of Exercise and Sport Science Matthew Gfeller Center University of North Carolina at Chapel Hill Chapel Hill, NC United States; 2 Department of Kinesiology and Sport Sciences Sport Neurotrauma Research Laboratory University of Miami Coral Gables, FL United States; 3 Department of Psychology and Neuroscience University of North Carolina at Chapel Hill Chapel Hill, NC United States; 4 Department of Biostatistics University of North Carolina at Chapel Hill Chapel Hill, NC United States; 5 Department of Exercise Science Elon University Elon, NC United States

**Keywords:** mild traumatic brain injury, concussion, autonomic nervous system, heart rate variability, military service members, collegiate athletes

## Abstract

**Background:**

Autonomic nervous system (ANS) dysregulation is increasingly linked to concussion. However, studies examining the relationship between concussion and ANS measures, such as heart rate (HR), HR variability (HRV), and pupillary light reflex (PLR), are limited by small sample sizes, inconsistent control of confounders, and methodological heterogeneity, thereby limiting clinical translation.

**Objective:**

The Measuring Autonomic Nervous System in Warfighters Following Exertion and Resting Stressors (ANSWERS) study aims to address these limitations by incorporating adequate sample sizes, using appropriate ANS recording protocols, and controlling for confounders.

**Methods:**

This 2-aim study integrates cross-sectional and longitudinal observational designs to clarify the relationship between ANS measures and commonly used symptom- and clinical performance–based assessments and to track how these measures change over time. In aim 1 (cross-sectional), we will evaluate differences and associations between ANS function and performance on standard postconcussion clinical performance and symptom measures in 150 participants (50 military service members [SMs] and 100 collegiate club athletes, analyzed separately), stratified by concussion history. Participants will complete resting and exertional ANS measures as well as a battery of cognitive, balance, vestibular, oculomotor, and symptom assessments. In aim 2 (longitudinal), we will track 50 athletes with concussion and 50 age-, sex-, and sport-matched controls using the same assessments across 3 time points: within 7 days of injury, at participant-reported symptom resolution, and 1 to 2 weeks following clearance for return to activity.

**Results:**

The Measuring ANSWERS study was funded in August 2023, with participant recruitment initiated in November 2023 and expected to continue through August 2027. As of July 2026, a total of 122 (48.8%) of the planned 250 participants have been enrolled, with data collection ongoing and study findings anticipated to be disseminated in late 2027.

**Conclusions:**

The Measuring ANSWERS study will provide a reproducible, standardized framework for assessing ANS function after concussion and may inform the integration of physiological markers into return-to-activity decision-making in both athlete and military populations.

**International Registered Report Identifier (IRRID):**

DERR1-10.2196/103400

## Introduction

### Background

Since 2000, there have been more than 432,000 diagnosed mild traumatic brain injuries (mTBIs), also known as concussions, in the US military [1]. In athletes, estimates suggest that 1.7 to 3 million concussions occur each year [2]. The current standard of practice after an athlete or service member (SM) sustains a concussion is a guided, stepwise approach to increasing activity until they can return to full, unrestricted activities. In the military, the progressive return to activity clinical recommendation (PRA-CR) is followed [3,4]. The PRA-CR uses a 6-stage approach with steps spanning early relative rest to unrestricted activity, each with structured guidance for progressive cognitive, physical, and vestibular activities. This design is analogous to the return-to-sport strategy described in the most recent international consensus statement on concussion in sport [5].

Return-to-activity guidelines are primarily guided by self-reported symptoms. Although self-report assessments are widely used, they may be susceptible to underreporting or may not fully capture physiological or functional recovery. In a critique of return to duty as an outcome metric, Cole et al [6] found that SMs often returned to duty before being medically cleared or were still experiencing clinically significant symptom levels even when they were medically cleared to return to duty. Returning to activity prior to true recovery may compromise force readiness in military SMs [6], increase the risk of musculoskeletal injury in athletes [7], and elevate the risk of adverse clinical outcomes, including second impact syndrome (SIS) and prolonged or persisting symptoms [8,9]. Ideally, return to unrestricted activity would be guided by objective indicators of physiological recovery.

Assessing the autonomic nervous system (ANS) is an emerging area of promise for objectively determining if an individual has physiologically recovered from a concussion. The ANS regulates involuntary physiological processes necessary for maintaining homeostasis, including cardiovascular and respiratory function [10]. Dysregulation of the ANS has been observed following concussion and possibly contributes to common postconcussion symptoms [11]. Measures reflecting autonomic regulation of cognitive, vestibular, and physiological processes, such as pupillary light reflex (PLR), baroreceptor sensitivity, heart rate (HR), and HR variability (HRV), are commonly used as indices of ANS function [12]. However, the overall evidence is mixed, with inconsistent associations between ANS measures and symptoms, particularly for resting measures, and much of the literature being derived from adolescent samples, with heterogeneous methodologies limiting cross-study comparisons [12-15]. Areas of emerging promise include assessing ANS function following exertion [16] and examining its relationship with postconcussion symptoms and results from common clinical measures [12,14,15].

A recent systematic review by Mercier et al [12] found that numerous studies demonstrated altered ANS functioning in individuals with concussion. However, most studies demonstrated a clear difference in ANS measures only during physiological challenges and not at rest. These findings suggest that challenge-based assessments may be necessary to detect subtle ANS dysfunction following concussion. High-intensity interval training (HIIT) may induce greater baroreceptor activation and subsequent cardiac vagal activity than steady-state exercise after 1 session [17] or following a short training program consisting of multiple HIIT sessions [18]. However, no studies have directly examined the link between ANS dysfunction and persisting postconcussion symptoms, highlighting the need for future research that explicitly tests these relationships across recovery stages [12]. The 39 studies included in the review used a range of autonomic metrics, with HR reported in 87% of studies and HRV reported in 46%. Measures such as baroreceptor sensitivity and PLR were used less frequently but may still offer added value. For example, prior research in military SMs found evidence of ANS dysfunction after concussion, as the rate of recovery during the PLR was slowed during the acute phase of concussion [19].

Mercier et al [12] identified additional gaps and methodological limitations in the existing literature on ANS dysfunction following concussion that must be addressed before future clinical applications. First, many studies failed to control for known confounders that influence ANS function, including medical conditions, medications, cardiorespiratory fitness, and menstrual cycle phase. Second, the duration of HR and HRV recordings varied widely, with several studies using ultrashort epochs (<4 minutes) despite evidence that reliable short-term HRV metrics require recordings of at least 4 minutes [20]. Third, most studies were potentially underpowered, with fewer than 25 participants per group, reducing confidence in between-group comparisons. Additional issues included inconsistent definitions of concussion, poorly characterized control groups (eg, those with unknown or positive concussion history), and inadequate reporting of sex and gender variables. Addressing these limitations will be essential for improving internal validity, reproducibility, generalizability, and the clinical translation of ANS-based measures in concussion research.

### Rationale and Objectives

The Measuring Autonomic Nervous System in Warfighters Following Exertion and Resting Stressors (ANSWERS) study directly addresses key methodological limitations identified in prior work and provides a reproducible framework for future clinical and interventional studies. More specifically, the study is designed to improve understanding of ANS dysfunction following concussion, with a focus on physiological mechanisms that may contribute to postconcussion symptoms and recovery trajectories. Both cross-sectional and prospective longitudinal observational designs are used to examine relationships among ANS function, commonly used clinical concussion assessments, and self-reported symptoms in 2 high-risk populations: college-aged athletes and active-duty military SMs. These groups share similar exposure risk profiles and return-to-activity decision frameworks, allowing findings to inform both return-to-play and return-to-duty practices. Study procedures include resting and exertional ANS assessments, standardized clinical performance measures, and symptom reporting.

This study design uses validated, physiologically grounded methods for assessing ANS function, with an emphasis on HRV during exertion, as most prior work has relied on resting measures despite growing evidence that physiological stress is necessary to reveal subtle ANS dysfunction. Empirically validated clinical performance and symptom measures were selected to reflect key functional domains commonly targeted in postconcussion assessments (eg, cognitive, vestibular, and psychological). Finally, we aim to incorporate sufficient data collection durations (eg, ≥5-minute resting and recovery periods for HRV) and account for key factors known to influence ANS metrics, including sex, menstrual cycle phase, physical activity levels, medical history, medication and substance use, and stimulant consumption.

### Study Aims

This study will examine associations between ANS function and clinical outcomes following concussion, as well as characterize recovery trajectories over time. Aim 1 will evaluate cross-sectional differences in ANS metrics, clinical performance measures, and symptom measures between individuals with and without a concussion history, adjusting for relevant demographic, medical, and behavioral covariates. Aim 2 will assess longitudinal changes in ANS metrics, clinical performance measures, and symptom outcomes in athletes with recent concussion compared to matched controls to determine whether physiological recovery parallels or diverges from clinical and symptom recovery. A detailed summary of the specific aims, hypotheses, variables, and analytic strategies is provided in Table 1.

**Table 1:** Summary of study aims, hypotheses, predictors, outcomes, and planned analyses.

	Aim 1	Aim 2
Specific aim	Examine the relationships between empirically supported ANS^a^ measures and commonly used, empirically validated concussion clinical performance measures	Develop an ANS-informed model of concussion recovery using longitudinally assessed ANS measures and clinical performance and symptom measures in athletes with recent concussion and age- and sport-matched controls
Hypothesis	ANS metrics will significantly differ between individuals with and without a history of concussion, similar to differences identified in clinical performance measures.	Athletes with a concussion will show changes in ANS metrics, clinical performance measures, and symptom trajectories over time relative to matched controls. Recovery trajectories in ANS metrics will mirror improvements in clinical performance measures and self-reported symptoms.
Independent variables	Concussion history	Group, time, and group×time interaction
Dependent variables	ANS metrics, clinical performance measures, and symptoms	ANS metrics, clinical performance measures, and symptoms
Planned analyses	Regression models adjusted for relevant demographic, clinical, and behavioral covariates	Mixed-effects models adjusted for relevant demographic, clinical, and behavioral covariates

^a^ANS: autonomic nervous system.

## Methods

### Overview

The study uses a prospective longitudinal observational cohort design, with a cross-sectional aim 1 and a longitudinal aim 2. Methods are described below in terms of the setting, participants, eligibility criteria, procedures, outcome measures, and planned analyses. The study design and reporting approach were informed by established methodological reporting guidance for observational studies and research protocols, and future reports of study findings will follow appropriate reporting standards for observational research.

### Study Setting

All data collection will occur on the campus of the University of North Carolina (UNC) at Chapel Hill.

### Participants

#### Aim 1 Participants

Aim 1 participants will be sampled from 2 populations: 50 active-duty military SMs and 100 UNC club sport athletes who participate in sports associated with a high risk for concussion. These populations were selected due to their elevated concussion risk and structured return-to-activity frameworks. Club sport athletes were selected rather than varsity athletes to maximize recruitment feasibility while maintaining ecological validity. Club sports at Division I institutions are highly competitive and include many contact and collision sports with elevated concussion risk. Nationally, there are >8.5 million collegiate club athletes in the United States, with approximately 45% reporting a history of at least 1 concussion, yet this population remains underrepresented in concussion research [21].

SMs and athletes will be classified as having a self-reported history of concussion (Hx+) or being “TBI naive” (ie, no self-reported history of concussion [Hx–]). We will selectively sample to ensure that we have at least 20 SMs and 40 athletes in each concussion history subgroup and approximately 50% male and 50% female participants in our club sport athlete group (±5%), recognizing that our military cohort will be predominantly male.

#### Aim 2 Participants

Aim 2 participants will include 50 UNC club sport athletes with concussion, as well as 50 age-, sex-, and sport-matched club athlete controls. Athletes are eligible if they have received a concussion diagnosis from an athletic trainer or campus health provider and are enrolled within 7 days of injury. Control participants will be identified from the sample of participants assessed in aim 1 or recruited from teammates of athletes enrolled after concussion and will be matched on club sport, age (±1 year), and sex.

### Inclusion and Exclusion Criteria

Participants are eligible to enroll if they are aged between 18 and 35 years, are active-duty US SMs (aim 1), or are UNC club sport athletes participating in a sport at high risk for concussion (aims 1 and 2). For this study, a “high-risk” sport is defined as one with an increased risk of concussion because it involves regular physical contact or collision (eg, rugby, ice hockey, lacrosse, and soccer), as well as sports involving high-velocity movements and a risk of falls from height (eg, gymnastics and cheerleading). This aligns with the classification of collision and high-contact sports reported in a recent epidemiological study of concussion in collegiate sports [22].

Participants are not eligible for this study if they have a history of moderate to severe brain injury or penetrating head injury, or a history of concussion in the last 12 months (excluding the recent concussion in athletes with concussion enrolled in aim 2). Other exclusionary criteria include a diagnosis of any major neurological, cardiac, or psychological disorder; untreated attention-deficit/hyperactivity disorder (ADHD); the presence of a heart pacemaker or other implanted electronic medical device; nonfunctional hearing; and severe visual deficits.

### Sample Size Justification

We conducted an a priori power analysis for the study design of aim 1, which includes 50 active-duty SMs and 100 club sport athletes. Each group is expected to have at least 40% of participants reporting a positive concussion history. Power analyses were developed for continuous ANS outcomes of interest using standardized mean differences (Cohen *d*), with mean HR serving as a representative continuous physiological outcome during study planning. Under these assumptions, the power to detect a medium effect size (*d*=0.50-0.60) ranged from 0.77 to 0.90, supporting the ability to detect moderate effects across continuous ANS outcomes.

For aim 2, we conducted an a priori power analysis based on a planned sample of 50 participants with concussion and 50 age-, sex-, and sport-matched controls. Using a 2-sided test with α=.05, we evaluated the minimum detectable effect sizes under varying assumptions of within-subject correlation (*r*=0.20-0.40) and power (80%-85%). Across these parameters, the minimum detectable effect size ranged from *d*=0.38 to *d*=0.46, indicating adequate power to detect medium effects.

### Overview of Study Procedures

Prior to participation, all individuals will provide informed consent via an electronic form to ensure that they understand the study procedures, potential risks and benefits, and participant rights. Institutional review board (IRB)–approved research personnel will review the study with participants, answer all questions, and witness the electronic signature.

#### Aim 1 Procedures

**Figure 1:**
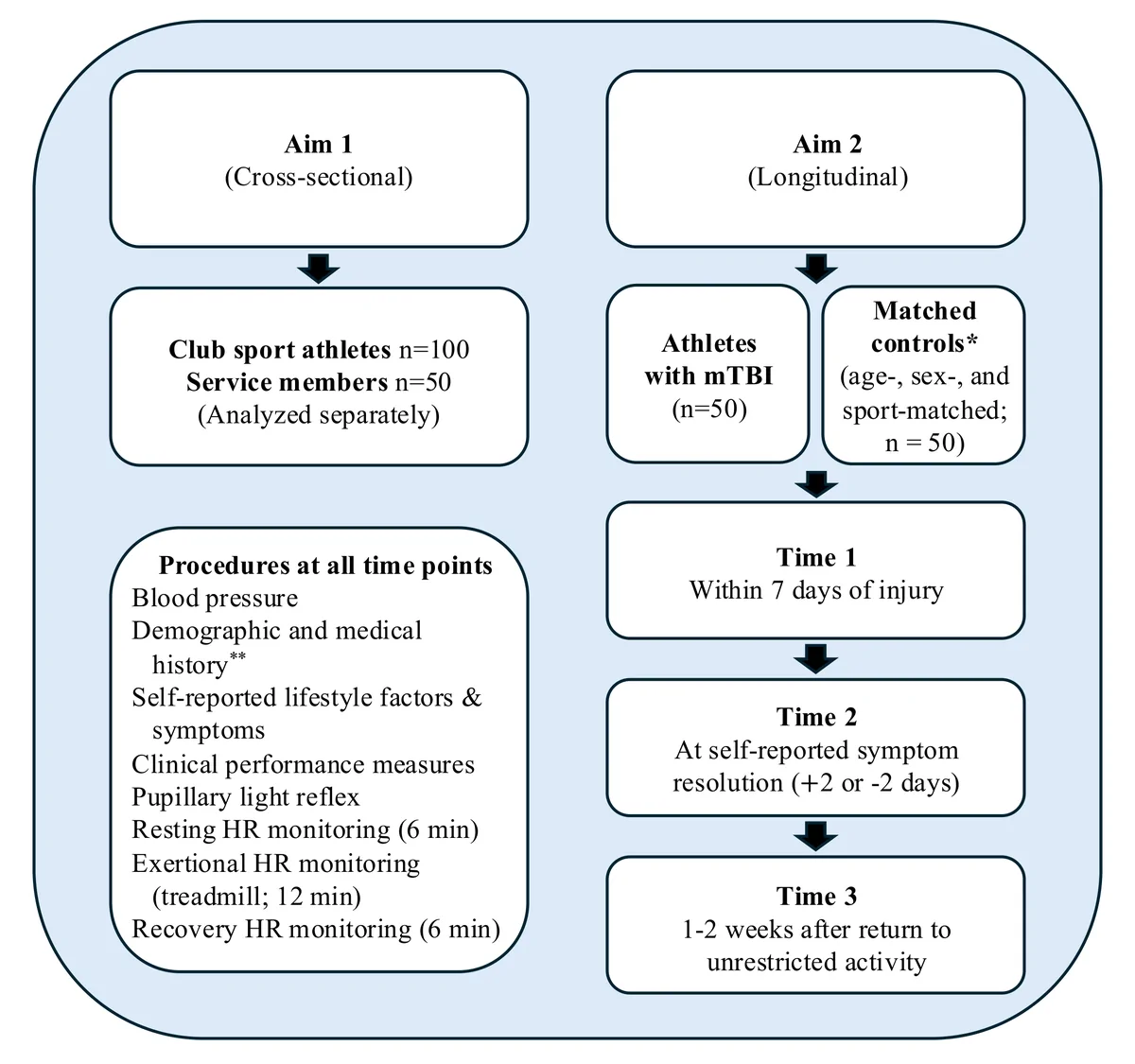
Study design, assessment timeline, and assessment procedures for aims 1 and 2. *Time 2 and time 3 assessments are scheduled to match those of the athlete with mild traumatic brain injuries (mTBIs). **Not administered at time 2 or time 3 of aim 2. HR: heart rate.

Aim 1 participants will complete a single in-person visit (approximately 90 minutes) consisting of a standardized battery of assessments (Table 2; Figure 1). Procedures include, in order of administration, blood pressure (BP) measurement, common postconcussion clinical performance measures, PLR assessment, completion of demographic and medical history questionnaires, a 6-minute resting HR recording, a standardized 12-minute treadmill exertion task with continuous HR monitoring, and a 6-minute recovery period with HR recording.

**Table 2:** Measures and associated outcome metrics.

	Measures used^a^	Domains	Primary outcome metrics
**Symptom measures**			
	Medical history questionnaire	Demographics and clinical covariates relevant to the autonomic nervous system function	Concussion history, current medications, current caffeine use, and menstrual cycle phase (female participants)
	Post-Concussion Symptom Scale^b^ [23]	Postconcussion symptoms	Total score (range 0-144)
	Patient Health Questionnaire–9^b^ [24,25]	Depression	Total score (range 0-27)
	Generalized Anxiety Disorder diagnostic tool–7^b^ [26]	Anxiety	Total score (range 0-21)
	Posttraumatic Stress Disorder Checklist^b^ [27]	Posttraumatic stress symptoms	Total score (range 0-80)
	Perceived Stress Scale [28]	Stress	Total score (range 0-40)
	National Institute on Drug Abuse–Tobacco, Alcohol, Prescription Medication, and Other Substance Use Tool [29]	Substance use	Past and current tobacco use (binary variable) and past and current average alcoholic drinks per week
	Pittsburgh Sleep Quality Index^b^ [30-32]	Sleep quality	Composite score (range 0-21)
	International Physical Activity Questionnaire [33]	Physical activity	Number of days and minutes per day of vigorous, moderate, and light physical activity, as well as time spent sitting
**Clinical performance measures**			
	Senaptec Sensory Station (Reaction Time and Eye-Hand Coordination subtests)	Simple RT^c^ and RTV^d^ and functional RT and RTV	Mean RT and trial-by-trial RT variability (milliseconds)
	Modified Balance Error Scoring System^b^ [34]	Postural stability	Total score across 3 trials (range 0-30)
	Y-Balance Test with and without visual occlusion [35,36]	Dynamic balance	Composite score for each visual condition (eyes open, low occlusion, and high occlusion)
	Vestibular/Ocular Motor Screening^b^ [37,38]	Vestibular and oculomotor function	Total posttest symptom score minus the total pretest symptom score and binary variable indicating if any symptom score increased by ≥2 points after testing
**Autonomic nervous system assessments**			
	Polar H10 Heart Rate Monitor	Heart rate and heart rate variability	Mean heart rate (beats per minute); heart rate variability time-domain metrics^c^: mean RR interval, root mean square of successive differences, SD of RR intervals (the latter two will serve as the primary heart rate variability metrics; all other variables will be evaluated as secondary or exploratory outcomes), percentage of successive RR interval differences >50 ms; and HRV frequency-domain metrics: total power, VLF^e^, LF^f^, HF^g^, normalized low-frequency power (LF/(total power−VLF), normalized high-frequency power (HF/(total power−VLF), and LF:HF ratio
	Automated sphygmomanometer	Blood pressure	Resting systolic and diastolic blood pressure (mm Hg)
	Pupillometer (PLR-3000)	Pupillary light reflex	Pupillary reaction latency, average constriction velocity, maximum constriction velocity, average dilation velocity, and time to reach 75% of the original diameter following peak constriction (all measured in ms or mm/ms)

^a^These measures will be used as symptom outcomes and/or covariates in statistical models, as appropriate.

^b^Measure recommended in the National Institute of Neurological Disorders and Stroke Sport-Related Concussion Common Data Element.

^c^RT: reaction time.

^d^RTV: reaction time variability.

^e^VLF: very low frequency power.

^f^LF: low frequency power.

^g^HF: high frequency power.

#### Aim 2 Procedures

Aim 2 participants will complete the same standardized assessment battery as that used in aim 1, in the same order, across 3 in-person visits (Table 2; Figure 1). Athletes who sustain a concussion will be assessed within 7 days of injury (visit 1), when they self-report being asymptomatic (±2 days; visit 2), and within 2 weeks of being medically cleared to return to unrestricted activity (visit 3). Visit 1 will include demographic and medical history questionnaires, clinical performance and symptom measures, and resting and exertional ANS assessments. Visits 2 and 3 will not repeat the demographic and medical history questionnaires but will repeat the clinical performance and symptom measures, along with the resting and exertional ANS assessments.

The matched control for each injured athlete will complete assessments at the same time points as their specific matched injured athlete.

### Assessments

#### Demographic, Medical History, and Self-Reported Symptoms

To account for individual and contextual factors known to influence physiological measures, participants will complete questionnaires assessing overall physical activity level, caffeine use, nicotine use, alcohol use, medication use, sleep duration on the night before testing, overall sleep quality, anxiety symptoms, posttraumatic stress symptoms, depressive symptoms, perceived stress, concussion history, and menstrual cycle phase (for female participants). Demographic and sport-related variables, including age, sex, and sport, will also be recorded. These variables will be considered as prespecified covariates or included in sensitivity analyses, as appropriate (refer to the Statistical Analysis section for details). Because the study is intended to characterize ANS responses under clinically relevant and ecologically valid testing conditions, these factors will be measured and analytically addressed rather than experimentally manipulated.

Refer to Table 2 for a list of demographic, medical history, and symptom measures and associated outcome metrics. Measure selection was guided by the National Institute of Neurological Disorders and Stroke (NINDS) Sport-Related Concussion Common Data Elements (CDE) recommendations. In addition to basic demographics, the medical history questionnaire captures traumatic brain injury (TBI) history, current medications, caffeine intake, and menstrual cycle phase (for female participants). TBI history is assessed using a questionnaire from previous work with Special Operations Forces combat soldiers [39,40], in which participants indicate if they have ever been diagnosed with a concussion by a clinician and report their lifetime concussion incidence (0-10). Symptom measures include validated and commonly used self-report instruments capturing concussion-related symptoms [23], as well as other mental health and psychosocial factors, such as depression [24], anxiety [26], stress and posttraumatic stress [27,28,41], substance use [29], sleep [30-32], and physical activity [33].

#### Clinical Performance Measures

Clinical performance measures were selected to capture core functional domains relevant to concussion using instruments with strong empirical support and widespread clinical and research use. These domains include reaction time and reaction time variability (via the intraindividual SD of trial-by-trial reaction time [42]), postural stability [34], dynamic balance [35,36], and vestibular and oculomotor function [37,38]. Metrics listed in the NINDS CDE recommendations were selected when available and appropriate for the study aims. Detailed task procedures, scoring metrics, and psychometric properties are provided in Table 2 and the corresponding references.

#### Measures of Autonomic Function

Refer to Table 2 for a list of ANS measures and their associated primary and secondary outcome metrics. ANS measures focus on 4 commonly used physiological proxies of ANS function: BP, HR, HRV, and PLR. Primary HRV outcomes will be the root mean square of successive differences (RMSSD) and the SD of RR intervals (SDNN), while the remaining HRV and other ANS metrics will be analyzed as secondary or exploratory outcomes.

Data will be collected using a standardized testing protocol that includes a seated resting period, a standardized treadmill exertion task, and a seated postexertion recovery period. Data acquisition procedures, equipment, task instructions, and processing methods will be consistent across participants. Although the time of day is not standardized, attempts will be made to schedule follow-up visits at approximately the same time, when feasible. The time of day for testing will be recorded and analytically addressed. Diastolic and systolic BP will be obtained from the brachial artery using an automated sphygmomanometer at the beginning of the data collection session. HR data will be measured (100 Hz sample rate) using Polar H10 Heart Rate Monitors (Polar Electro Oy) before, during, and after a standardized treadmill exertional task (as described below).

To ensure adequate recording durations for analysis, a minimum of 5-minute recordings will be used for the resting, exertion, and recovery phases. Participants will be instructed to remain seated quietly with minimal movement during the resting and recovery recordings and to breathe normally throughout data collection. HR and HRV metrics will be extracted using Kubios HRV Premium (version 3.5.0; Kubios Ltd) software, which applies artifact correction and detrending algorithms as described by Tarvainen et al [43]. PLR will be assessed using a handheld pupillometer (PLR-3000; NeurOptics Inc). Three valid trials will be performed for each eye, with a 30-second recovery period between each trial to ensure that no differences exist between eyes.

#### Treadmill Task

An electric treadmill will be used with a standardized high-intensity exercise protocol to stress the ANS to a greater extent than steady-state exercise [44,45]. Prior to engaging in the treadmill task, participants will complete a 6-minute seated rest period to obtain resting HR metrics. They will then complete a 12-minute treadmill running protocol that alternates between slow and fast treadmill running speeds based on 60% and 90% of the superior category (90th percentile) for aerobic capacity [44,46,47]. Participants will complete a 2-minute warm-up at a moderate-intensity speed, followed by 30-second intervals (1:1 ratio) alternating between high- and moderate-intensity speeds for 10 minutes.

ANS responses following HIIT sessions with 30-second intervals were similar to those following sessions with 15- and 60-second intervals [48]. Target speeds differ by sex (male participants: 3.8 m/s and 2.5 m/s; female participants: 3.1 m/s and 2.0 m/s) [49]. Previous work by members of our research team has found that this assessment resulted in a submaximal HR (approximately 168 beats per minute) following the aerobic component, which was more reliable and precise (intraclass correlation coefficient [ICC]=0.723, 95% CI 0.566-0.824) than the maximal HR (approximately 175 beats per minute) following other commonly used exertion protocols [50]. Thus, this task more effectively controls exercise intensity, a core exercise prescription factor that can influence postexercise HRV measurements, than do steady-state or maximal exercise assessments. After completing the treadmill task, participants will engage in a 6-minute rest or recovery period during which HR will be recorded.

#### Safety Considerations

This study involves participants in the postacute phase of concussion and includes physical and psychological assessments that could identify medical or mental health risk. Potential risks include transient symptoms (eg, dizziness, nausea, and exertional discomfort) during exertional tasks and an increased fall risk during balance testing. All procedures will be conducted under trained supervision with continuous monitoring, predefined stopping criteria for symptom exacerbation or participant discomfort, and established referral pathways for any psychological safety concerns identified during symptom screening. Medical providers and athletic training staff will be available as needed to ensure participant safety.

### Statistical Analysis

#### Analyses of Aim 1 Data

Club sport athletes and SMs will be analyzed separately. Primary analyses will compare RMSSD and SDNN between participants with and without concussion history, with emphasis on changes from rest to postexertion recovery. RMSSD and SDNN were selected based on emerging evidence of their reliability and sensitivity to physiological changes following concussion [12,15]. Additional HRV metrics, along with HR, BP, and PLR measures (Table 2), will be analyzed as secondary outcomes in a similar manner. The analytic approach will be tailored to the outcome type. Continuous outcomes will be analyzed using linear regression models, and binary outcomes will be analyzed using logistic regression models. Time-to-event outcomes derived from PLR will be analyzed using Cox proportional hazards models.

Across all models, prior concussion history (Hx+ vs Hx−) will be the primary exposure. Models will adjust for prespecified demographic and clinical covariates known to influence ANS function and recovery (Table 2). Candidate covariates include age; sex; physical activity; sleep quality; medication use; caffeine use; nicotine use; and other demographic, behavioral, and clinical factors included in Table 2. Because not all covariates are expected to be relevant to every outcome, covariates will be selected for individual models based on the outcome under investigation and scientific rationale, although sex will be retained in all candidate models. To account for heterogeneity in injury exposure, exploratory analyses will examine dose-response relationships using indicators of prior concussion frequency and recency (eg, the number of prior concussions categorized as 0, 1-2, or 3 and time since the most recent injury), modeled either as covariates or in stratified analyses, as appropriate.

Effect modification will be explored in secondary analyses by adding a limited set of prespecified clinically relevant interaction terms (eg, age×BMI and depression×anxiety). To limit the risk of type I error resulting from multiple testing of secondary outcomes, false discovery rate (FDR) procedures will be applied within outcome domains. Missing data will be handled using standard imputation methods (eg, multiple imputation [51] and multivariate imputation by chained equations [52]).

#### Analyses of Aim 2 Data

To evaluate whether concussion (vs control) is associated with differential longitudinal changes in ANS metrics, clinical performance, and symptom outcomes, we will fit a series of mixed-effects models, 1 per outcome. Primary ANS outcomes will be RMSSD and SDNN. Symptom measures and clinical performance measures collected at each visit will be evaluated, while additional ANS metrics will be included in secondary analytic models (Table 2). Each model will include fixed effects for group (concussion vs control), time (visit 1, visit 2, and visit 3), and the group-by-time interaction to test for between-group differences in trajectories. Time will be modeled as a categorical variable to reflect clinically meaningful recovery stages (acute injury, symptom resolution, and return to unrestricted activity), consistent with current return-to-activity frameworks.

Models will additionally adjust for prespecified demographic, clinical, and behavioral covariates known to influence ANS function and recovery (Table 2). Candidate covariates will be selected a priori for individual models based on biological plausibility, the outcome under investigation, and the existing literature. Sex will be retained in all models. A participant-level random effect (random intercept) will be included to account for the correlation among within-subject repeated measurements. A compound symmetry working covariance structure will be assumed for repeated measures.

Given the anticipated sample size and the potential for convergence or boundary issues, we will implement a prespecified fallback strategy. If a mixed-effects model fails to converge for a given outcome, we will instead fit outcome-appropriate regression models at each visit separately (linear regression for continuous outcomes and logistic regression for binary outcomes) to estimate cross-sectional concussion-vs-control differences at each time point, acknowledging the reduced efficiency relative to a single longitudinal model.

### Ethical Considerations

Ethics approval for the study was provided by the IRB at UNC (23-1012 and 23-2383) and the US Army Medical Research and Development Command’s (USAMRDC) Office of Human Research Oversight (OHRO). Participant data will be deidentified and assigned unique study IDs. All deidentified behavioral, clinical, and physiological data will be stored and managed using secure, encrypted platforms consistent with UNC and Department of Defense data protection requirements. Identifiable information will be stored separately from research data on secure, access-controlled institutional servers at UNC. Only authorized study personnel will have access to identifiable information. In alignment with federal public access mandates and Congressionally Directed Medical Research Programs (CDMRP) data-sharing requirements, deidentified participant-level data will be managed, preserved, and shared in accordance with applicable sponsor policies and institutional requirements to facilitate appropriate secondary research use.

## Results

The Measuring ANSWERS study was funded in August 2023. Participant recruitment began in November 2023. As of July 2026, a total of 122 (48.8%) of the planned 250 participants have been enrolled across the study aims. Data collection is ongoing and is anticipated to be completed by August 2027. Primary analyses for the study aims have not yet been initiated. Primary analyses will begin following completion of data collection for each study aim, and findings will be disseminated as soon as feasible thereafter. Dissemination plans include presentation of findings at national scientific meetings and publication of the primary study findings in peer-reviewed journals.

## Discussion

### Anticipated Findings

The Measuring ANSWERS study is designed to determine whether ANS measures obtained at rest and following exertion provide clinically meaningful information beyond current symptom- and performance-based concussion assessments. Specifically, the study addresses critical gaps in the understanding of ANS dysfunction following concussion by integrating physiological, clinical, and symptom-based assessments within complementary cross-sectional and longitudinal study designs while incorporating validated outcome measures, standardized recording protocols, and systematic consideration of known confounders. This approach builds upon a growing body of literature linking markers of ANS dysfunction to concussion [12,15] while addressing key methodological gaps and the limited evidence connecting postconcussion physiological changes to clinically actionable outcomes [12].

Through 2 complementary aims, we will examine both cross-sectional and longitudinal relationships between ANS function and clinical recovery. Aim 1 evaluates associations between physiological markers (eg, HRV, PLR, and BP) and commonly used postconcussion clinical performance and symptom measures in 2 high-risk populations—military SMs and collegiate athletes—broadening the applicability of the findings across operational and sport settings. We hypothesize that ANS measures obtained at rest and following exertion will distinguish individuals with and without a concussion history and demonstrate meaningful associations with commonly used clinical performance and symptom measures.

Aim 2 follows athletes with a concussion and age-, sex-, and sport-matched controls across 3 time points to determine whether physiological recovery parallels or diverges from clinical recovery and symptom resolution. We hypothesize that ANS function, particularly following exertion, will differ between the concussion and control groups across the time points and will demonstrate meaningful associations with commonly used clinical performance and symptom measures over the course of recovery. If confirmed, these findings would extend prior work suggesting that physiological monitoring before, during, and after exercise may identify persistent autonomic dysfunction during concussion recovery and inform return-to-activity decision-making [53]. Accordingly, this study is designed to determine whether ANS measures provide clinically relevant information beyond standard symptom reports and performance-based assessments. If ANS recovery lags behind symptom and clinical recovery, this may indicate the presence of residual physiological dysfunction not captured by current assessment approaches. The functional significance of such differences remains unclear and warrants further investigation, particularly with respect to their potential impacts on real-world performance. If confirmed, such findings would support the integration of exertion-based ANS measures into return-to-activity decision-making frameworks (eg, PRA-CR).

### Potential Challenges and Mitigation Efforts

There are several anticipated challenges to executing this study. Recruitment and retention, particularly in the longitudinal arm (aim 2), may be difficult among collegiate athletes. We are leveraging close collaboration with UNC club sports, athletic trainers, and sports medicine staff to increase recruitment and minimize attrition. The heterogeneity of injury characteristics, particularly in the aim 1 Hx+ cohort, could present challenges, as is common in concussion research. The analytic plan addresses this through covariate adjustment and, where the sample size permits, exploratory stratified analyses. Finally, ANS measures are sensitive to numerous physiological and contextual factors (eg, sleep, caffeine intake, hydration, and recent activity). Thus, relevant covariates will be measured and modeled, and analyses will emphasize within-subject change in aim 2. Future analyses may explore multivariate or data-driven approaches (eg, clustering or composite indices) to identify ANS phenotypes associated with recovery trajectories.

### Conclusions

The Measuring ANSWERS study aims to advance the current state of the field through the evaluation of ANS function after concussion using large sample sizes, validated clinical performance measures, and well-established physiological assessments. By comparing individuals with and without a history of concussion and tracking recovery trajectories over time, we aim to determine whether physiological recovery mirrors improvements in symptoms and clinical performance or whether it identifies residual deficits that current assessments may miss.

If successful, this study will contribute important evidence regarding the relevance of ANS markers in postconcussion care and may support the integration of more objective physiological indicators into return-to-activity protocols. Ultimately, this work may help shape safer, more personalized recovery timelines for individuals with concussion and improve clinical decision-making in both athlete and military populations.

## Data Availability

Deidentified datasets will be prepared for sharing in accordance with funding agency requirements.

## Appendix

Multimedia Appendix 1 Peer review report by the Office of the Assistant Secretary of Defense for Health Affairs, through the Traumatic Brain Injury and Psychological Health Research Program under Award No. HT94252310642.

## Abbreviations

ADHD: attention-deficit/hyperactivity disorder
ANS: autonomic nervous system
Measuring ANSWERS: Measuring Autonomic Nervous System in Warfighters Following Exertion and Resting Stressors
BP: blood pressure
CDE: Common Data Elements
CDMRP: Congressionally Directed Medical Research Programs
FDR: false discovery rate
HIIT: high-intensity interval training
HR: heart rate
HRV: heart rate variability
Hx+: self-reported history of concussion
Hx−: no self-reported history of concussion
ICC: intraclass correlation coefficient
IRB: institutional review board
mTBI: mild traumatic brain injury
NINDS: National Institute of Neurological Disorders and Stroke
OHRO: Office of Human Research Oversight
PLR: pupillary light reflex
PRA-CR: progressive return to activity clinical recommendation
RMSSD: root mean square of successive differences
SDNN: SD of RR intervals
SIS: second impact syndrome
SM: service member
TBI: traumatic brain injury
UNC: University of North Carolina
USAMRDC: US Army Medical Research and Development Command
