# Supplementary material for: Autonomic Nervous System Responses to Rest and Exertion Following Concussion (the Measuring ANSWERS Study): Protocol for a 2-Aim Cross-Sectional and Longitudinal Observational Study
Source: JMIR Res Protoc. 2026 Aug 14;15:e103400. doi: 10.2196/103400 (PMC13525365; doi:10.2196/103400)
Supplement: Supplementary file 1 [file resprot_v15i1e103400_app1.pdf]

Department of Defense
U.S. Army Medical Research and Development Command
Congressionally Directed Medical Research Programs
Fiscal Year 2022 Traumatic Brain Injury and Psychological Health Research Program
Translational Research Award - Human Subjects
Peer Review Summary Statement

CDMRP Log Number: TP220063
Grants.gov ID Number: GRANT13712324
Review Panel: Detection, Diagnosis and Prognosis - 3
Discussion Period: 11/17/2022-11/18/2022

Project Duration: 36 months
Budget Requested: $1,555,000
Direct Costs: $1,000,000
Indirect Costs: $555,000

Title: Measuring Autonomic Nervous System in Warfighters Following Exertion and Resting Stressors (Measuring ANSWERS)
Principal Investigator: Wesley Cole
Performing Organization: North Carolina at Chapel Hill, University of
Contracting Organization: North Carolina at Chapel Hill, University of

OVERVIEW

The Principal Investigator (PI) of this application proposes the objective of the project is to link physiologic functioning to commonly used mild traumatic brain injury (mTBI) symptom and clinical measures, as well as to ecologically valid functional assessments, to increase confidence in clinical tools and determinations regarding safe return to full unrestricted activity and duty. The project's specific aims are (1a) to determine the effect of mTBI history on autonomic nervous system (ANS) functioning and postconcussion assessment measures, while controlling for common ANS confounds; (1b) identify impact of ANS dysregulation on real-world functional assessments; and (2) develop an ANS model for mTBI evaluation by tracking ANS functioning over time after injury, while simultaneously collecting commonly used clinical and symptom assessments.

	**Average Score**	**Standard Deviation**
**Overall Evaluation**<br>*Rating Scale: 1.0 (highest merit) to 5.0 (lowest merit)*	2.1<br>**(Good)**	0.2
**Criteria**<br>*Rating Scale: 10 (highest merit) to 1 (lowest merit)*	**Average Score**	
**Research Strategy and Feasibility**	6.0	
**Clinical Strategy**	6.0	
**Impact and Relevance to Military Health**	7.8	
**Personnel**	9.0	
**Ethical Considerations**	7.2	
**Statistical Plan and Data Analysis**	5.8	
**Data and Research Resources Sharing Plan**	7.0	
**Transition Plan**	7.2	

SCORED CRITERIA

Research Strategy and Feasibility
Average Score: 6.0

Scientist Reviewer A

The proposed study has 2 major aims. Specific Aim 1 is focused on linking ANS functioning to current mTBI measures and will use a cross sectional design with active duty service members and college athletes. Test subjects will complete a battery of TBI and ANS assessments. A virtual reality (VR) assessment will be used to characterize participants' behavioral outcomes. The analytic approach for Aim 1 is focused on a variety of regression models to assess the relationship between various ANS and outcome measures and mTBI history. Specific Aim 2 is focused on assessing ANS functioning after mTBI and will utilize a prospective cohort of athletes with a recent mTBI and matched healthy control cohort. All participants will complete a comprehensive set of mTBI and ANS assessments as well as a VR assessment. Assessments will be done at 3 different time points for both cohorts. The analytic approach for Aim 2 is focused on mixed effect models to assess change over time in the various measures.

Strengths: The proposed research addresses fiscal year 2022 Traumatic Brain Injury and Psychological Health Research Program (FY22 TBIPHRP) focus area 1a (increase understanding of biological underpinnings of TBI and risk for prolonged symptoms) by seeking to understand ANS dysregulation after mTBI. The literature review and scientific rationale provide adequate justification for the overall approach and assessment of ANS measures. The applicants note that current return to duty and return to play decisions typically rely upon self-reported symptoms, return to duty is often ill-defined, and assessments are not typically ecologically valid. The applicants have appropriately called out methodological shortcomings of prior work that they will address (eg, appropriate recording length for resting heart rate).

The purpose, objective, and specific aims are crisply described and align well with the Statement of Work (SOW.) The applicants have thoroughly described the design and methods to address each of the 2 aims. The first aim will leverage a cross sectional design with active duty service members and college athletes. All will complete a battery of TBI and ANS assessments and will complete a virtual reality assessment to characterize behavioral outcomes. The second aim will utilize a prospective cohort of athletes with a recent mTBI and matched healthy control cohort. All participants will complete a comprehensive set of mTBI and ANS assessments as well as a VR assessment. Assessments will be done at 3 different time points for both cohorts. These methods are appropriate for the project goals. The proposed research is feasible and can be completed within the amount of time proposed.

The proposed enrollment on the basis of various demographic variables is appropriate given the populations of interest. Women will be included as participants in the non-service member populations. The service member population is a Special Forces one composed mostly of men. Women and minorities will not be excluded from participation.

Weaknesses: Some moderate weaknesses were noted. While a literature review is included to justify the proposed approach, it is somewhat brief—more justification and detail could have been provided. The application is generally light on descriptions of potential problems or risks. There are very few noted explicitly; the team instead presents their confidence in successfully executing all aspects of the proposed research. The SOW does not appear to include government IRB approval, and as such there may not be enough time in the schedule to submit and receive approval before recruitment can commence. They

expect University of North Carolina at Chapel Hill (UNC) approval in Month 4, but do not include a similar milestone for government IRB approval.

Scientist Reviewer B

Strengths: The proposed research adheres to the CDMRP FY22 TBIPHRP focus area 1a by aiming to improve the understanding of biological underpinnings of TBI and individual risk for prolonged symptoms. The proposal seeks to learn more about the ANS and concussion recovery with the ultimate goal of improving clinical decision-making following concussion. The applicants do a nice job of highlighting the existing gaps in the research and how their proposed project will make a significant contribution to filling these gaps. They acknowledge that return to duty is not well-defined in the military literature and is an important consideration and one that could be significantly improved by the results of this project, if successful. Similarly, it is well-defined how the methodology of this proposal improves upon the methodology of previous studies in the field. The scientific rationale behind the study proposal is strong, and it is evident how the results of this study may potentially make a significant contribution to both military and sports medicine. The aims and hypotheses are clearly defined and easy to follow. The inclusion of Table 1 is a well thought out addition. The aims are appropriate and suitable for addressing the overarching research questions.

There is a strong community relationship between the UNC Matthew Gfeller Center and the US Army Special Operations Command (USASOC) that will enable ample recruitment opportunity for the targeted participants. This existing research collaboration is the foundation for the proposed aims. The existing pipeline will benefit the study by increasing the likelihood that the proposed aims can be completed within the proposed time line. Additionally, the proposed study aims are feasible given the expertise of the research team and the UNC facilities. Due to the existing research infrastructure already in place at UNC, there is no reason to believe that the proposed aims and assessments cannot be completed in the provided time line.

The study design and outcome measures are appropriate to answer the proposed research questions. The applicants do a nice job of including separate tables for each of the outcome measure categories, which helps significantly with the clarity of the proposal. The physiologic and clinical outcome measures that have been proposed are well established in the existing literature and well-suited to provide a comprehensive assessment of physical and mental functioning following mTBI. The inclusion of the VR task to provide an ecologically valid recovery scenario is an exciting, novel use of the tool. Finally, the proposal contains an appropriate inclusion of women and minorities, and it is clearly stated that no participants will be excluded based on race or sex.

Weaknesses: The proposal provides a lot of evidence of ANS dysfunction following concussion, but there are insufficient examples of specifically what dysfunction the applicants are referring to. This is a minor weakness. The hypotheses between Aim 1 and Aim 2 contradict each other. The applicants hypothesize that they expect to see clinically meaningful differences in ANS functioning in those participants with a history of mTBI compared to those with no history. However, the applicants state that they expect no statistically significant differences between the mTBI and control participants. It is unclear why the applicants would not expect to see differences between the mTBI and control groups but would expect to see them in participants with a history of mTBI. This is a moderate weakness. In the SOW, Specific Aim 1 is divided up into 1a (determine the effect of mTBI history on ANS functioning and postconcussion assessment measures while controlling for common ANS confounds) and 1b (identify impact of ANS dysregulation on real-world functional assessments); however Table 1 does not sufficiently explain this component of the design. Aim 1 is not divided into a and b components in Table 1, but there are 1a and 1b hypotheses. Aim 1 does not appear to be divided into 2 separate components throughout the rest of the research strategy document, and this is a moderate weakness.

There is insufficient clarity surrounding the sport types involved on the civilian side. The "Participants Aim 1" section states that club athletes from collision sports will be included, but not all the sport examples listed are collision sports, and the inclusion criteria do not specify only collision sport club athletes. This is a minor weakness. It is unclear if the history of mTBI group includes only a history of actual diagnosed mTBIs or if this will also include participants who potentially had mTBIs that were unreported or not officially diagnosed. This is a minor weakness.

There is inconsistency in the outcome measures when it comes to gait assessment. Under the "Measures Aim 1" section, gait is included as a common clinical measure that will be assessed in the study, but there is no inclusion of gait or tandem gait in Table 3 (clinical evaluations and associated outcome metrics). There also appears to be no gait assessment included or described in the "Clinical Measures" section of Aim 1, but tandem gait is listed under the procedures of Aim 2. It is unclear whether tandem gait is being included as one of the clinical measures. This is a moderate weakness. There is inconsistency in the description of the guided treadmill task. Under the "ANS Measures" section, it states there will be a 6-minute seated rest period prior to starting the treadmill protocol and after completing the treadmill task. However, in the "Procedures Aim 1" section, it states the initial and recovery rest periods will be 5 minutes. This is a minor weakness.

There is limited inclusion of preliminary data to support the feasibility of the project. The ANS and clinical measures are well established in the literature, but there are insufficient preliminary data for the VR task. This is a minor weakness. There is minimal discussion of the limitations or potential pitfalls of the proposed project. There are also limited alternative approaches included if the proposed methodology fails at any point. This is a significant weakness.

Clinical Strategy
Average Score: 6.0

Scientist Reviewer A

Strengths: The application contains a number of moderate strengths. The scientific rationale for the evaluation of ANS measures in mTBI is well-described and compelling. Both active duty service members and college athletes will be recruited. The team has appropriate access to these populations. The inclusion and exclusion criteria are different for the 2 aims. Participants for Aim 1 are to be healthy and have not had an mTBI within the last year. Participants in Aim 2 must be within 3 days of an mTBI; controls are age- and sex-matched. Exclusion criteria are generally appropriate to ensure that participants can complete the research activities safely and do not have confounding illnesses that would impact the validity of the ANS measures. The team has presented an enrollment schedule for participants that seems reasonable. The team has proposed data collection for a variety of clinical, physiological, and performance metrics to answer Aims 1 and 2. These are comprehensive and appropriate given the aims. The proposed enrollment on the basis of various demographics is appropriate and consistent with the service member and college athlete populations of interest.

Weaknesses: Several moderate weaknesses were noted. There is a significant disconnect between the inclusion/exclusion criteria listed in the Clinical Strategy section and the project narrative. The project narrative notes that for Aim 1, participants will be classified as those with and without mTBI history. Yet, the exclusion criteria for Aim 1 note that a history of mTBI in the last 12 months is an exclusionary factor, and there is no mention of an mTBI history in the inclusion criteria, which would seem necessary to meet the objective of Aim 1. As currently worded, it is implied that an mTBI greater than 1 year ago is the inclusion criterion for some participants, but this is not clear. Given the objectives of this aim, this is a critical issue and needs to be clarified to have confidence that an appropriate number of individuals with and without mTBI will be included.

An additional weakness noted is that the team does not identify potential barriers to accrual/retention. They note in the project narrative that they have successfully collected data from thousands of participants over the years but provide insufficient details about the reasonableness of their proposed quarterly enrollment table. This is particularly salient for the service member population, as service members can be difficult to both recruit and retain given their active duty status.

Scientist Reviewer B

Strengths: The rationale of the study is clear, and the applicants have successfully addressed how the study design will fill existing gaps in the research. There is appropriate inclusion of women and minorities in the study, and the applicants explicitly state that no participants will be excluded based on race, ethnicity, or sex. The proposed enrollment is adequate for the study design and, if successful, will allow study recruitment to progress and reach the target numbers by the end of the time line. There is an existing pipeline in place between the USASOC and UNC that will make recruitment of service members from Fort Bragg feasible for the study. The Matthew Gfeller Center at UNC also has a history of successfully recruiting sport club athletes as research participants, so the targeted population is easily accessible. The proposed study assessments (clinical, physiological, and virtual reality) are relevant to the study objectives and will adequately answer the research questions if the study is successful. The screening and informed consent procedures are clearly defined and appropriate for the proposed study. The potential risks and benefits of the study are clearly defined and appropriate for the proposed study.

Weaknesses: There is sparse clarification in the exclusion criteria of whether the mTBI had to be diagnosed, or if it also applies to undiagnosed, unreported, or suspected but not diagnosed mTBI. This is a minor weakness. There is limited discussion of potential barriers to successful study recruitment. There is limited discussed how the mTBI participant recruitment time line will be changed if necessary (eg, if it becomes difficult to get club sport athletes in to participate in the study within 3 days of their mTBI). This is a moderate weakness. It is unclear what the exclusion criteria consider to be a psychological disorder. It is unclear if anyone who has been diagnosed with anxiety, depression, or attention deficit disorder will be eligible. This is a minor weakness. There is insufficient rationale for why only undergraduate students are being included given that the age range is so large. This is a minor weakness.

Impact and Relevance to Military Health
Average Score: 7.8

Scientist Reviewer A

Strengths: The application contains several major strengths related to impact and relevance to military health. The goals of the proposed research are to identify links between ANS measures and commonly used mTBI evaluation measures and build an ANS model for mTBI recovery. Both will inform better and more objective return to duty/play decisions in military and civilian populations and are directly responsive to current challenges in standard of care and decision making after mTBI. Successful realization of both aims could lead to positive impacts on warfighter health and force readiness. As noted by the applicants, current standard of care in making return to duty/play decisions relies largely on subjective self-report measures. Objective indicators would greatly improve the assessment process.

Weaknesses: One noted moderate weakness is the general lack of a discussion about potential issues that may limit the impact of the proposed research. The applicants do have a record of successful execution of similar research, but really do not denote any potential issues, and there likely are several.

Scientist Reviewer B

Strengths: The proposed project clearly aligns with the goals of the funding mechanism, which include understanding the risk, protective, and biological factors contributing to an individual's vulnerability to, response to, and long-term outcomes of TBI. Furthermore, the proposal has the potential to make a significant impact in the recovery of service members following TBI. If successful, the findings in this study have the potential to improve the Department of Defense's progressive return to activity clinical recommendations and ensure that service members are not being returned to duty prematurely. The proposal is responsive to the needs of both military and civilian populations, as it will bridge the gap of understanding how "clinical recovery" from TBI translates into real world scenarios.

Weaknesses: There are sparse references included in the background section of the impact statement. This is a minor weakness. There is limited discussion of potential issues that may limit the impact of the proposed research. This is a moderate weakness. There is limited discussion of the potential long-range benefits of the proposed project on military health. This is a minor weakness.

Consumer Reviewer

Strengths: This could greatly improve the standard of care with improved clinical decision making, in the context of an aggressively evolving fighting force prioritizing readiness and lethality. This project's long range goal is to improve the current assessment using the marker of physiologic recovery, which could be a more accurate indicator for soldiers to return to duty. This proposal doesn't have any obstacles. It is very straightforward in finding an assessment tool to better warfighter standard of care. This is a moderate strength.

Weaknesses: There is insufficient discussion about how delays in the research would impact the proposed research.

Personnel
Average Score: 9.0

Scientist Reviewer A

Wesley Cole received his PhD in clinical psychology from the University of South Carolina in 2006. He completed a postdoc in neuropsychology at Johns Hopkins following completion of his PhD. He currently is a research associate professor in the Department of Exercise and Sport Science and neuropsychologist for the Transforming Health in Veterans (THRIVE) program at UNC.

Strengths: The composition of the team is excellent. Dr Cole, Dr Jason Mihalik, Dr Johna Register-Mihalik, and Dr Kane all have appropriate background and expertise in mTBI, neuropsychological measures, and/or pertinent experience with veterans and active duty service personnel. The team has an excellent publication and funding track record for research of similar domain and scope. Dr Kane served in the US Army as both a soldier and physician and has had several command-level roles in the military. He will bring excellent military expertise and experience to the proposed research. The levels of effort by the PI and key personnel are adequate and appropriate to allow for successful completion of the proposed research.

Weaknesses: No weaknesses were noted.

Scientist Reviewer B

Strengths: The proposed study team is strong and well-versed in the science of TBI on both the military and civilian side. The PI, Dr Cole, is a neuropsychologist with extensive experience working with military service members. The team is strengthened by the inclusion of Dr Zou in the biostatistician role. Dr Register-Mihalik and Dr Mihalik are renowned leaders in the field of sport and military concussion. Dr Kiefer has previously served as an investigator on funded proposals related to VR assessment, which plays a key role in this proposal. Dr Kane is a physician with extensive experience working with military personnel.

Weaknesses: No weaknesses were noted.

Ethical Considerations
Average Score: 7.2

Scientist Reviewer A

Strengths: The application contains several major strengths. The targeted populations of service members and athletes for Aim 1 and athletes within 3 days of an mTBI and matched healthy controls for Aim 2 stand to benefit from a better understanding of the relationship between ANS function measures and postconcussion assessment measures, and tracking of functioning over time. Results will inform updates to existing assessment batteries and return to play/duty assessments.

Risk has been appropriately minimized, and safety monitoring and reporting are appropriate. Participation risks are minimal, and participants will be closely monitored during the physical and VR tasks. Required reporting of events is detailed and appropriate. The privacy and confidentiality of study records are appropriately considered. Personally identifiable information will be appropriately protected, both electronically and for any paper records.

The informed consent process is appropriate; the consent form will be reviewed thoroughly with potential participants, and they will have ample time to review and decide whether or not to participate. The applicants have noted that special considerations will be in place from USASOC service members if there are protocols that prohibit their personally identifiable information to be included on consent documents. The applicants have additionally noted that procedures will be in place to ensure that commanding officers, coaches, and team members will not be present during the consent process to minimize coercion.

Weaknesses: One weakness noted in the consent form is that monetary compensation is offered as a benefit. That is not appropriate and should not be included.

Scientist Reviewer B

Strengths: The proposed project has the potential to significantly benefit both the military and civilian populations who are recovering from mTBI. The service members and club sport athletes who participate in the research will benefit from the knowledge to be gained from this study, if successful, as it will improve the understanding of autonomic functioning following concussion and potentially advance clinical decision-making during recovery. The risk level of the proposed research is minimal, and the risks are clearly detailed along with solutions to avoid them. There is a clear course of action if adverse events occur during the study, and 10% of participants reporting adverse events has been included as the threshold for study termination. Privacy and confidentiality considerations have been clearly defined and are adequate for the proposed study. The described informed consent process is appropriate for the

proposed study, and the applicants have included extra considerations for the informed consent of the service members who cannot provide identifying information on consent forms.

Weaknesses: Compensation is included as a benefit in the study, which is not appropriate. There may also be ethical considerations surrounding the lack of clarity in the inclusion/exclusion criteria.

Bioethicist Reviewer

Strengths: Moderate strengths include that deidentified data will be used, but subjects have the option of having their information released if medically relevant or otherwise beneficial information is discovered during the course of the study. The major strengths of the application include that the consent process is appropriate given the population and there is an appropriate safety plan given the questions subjects will answer, mitigating some risk of the study. Heart rate monitoring will also take place given the activities of the study. Study stopping considerations are appropriate.

Weaknesses: Moderate weaknesses of the application include that compensation to subjects should not be listed as a benefit of involvement. There also should be alternative compensation of service member subjects. Adverse events will be reported to study staff and then possibly to the PI. It is unclear who will actually do the reporting. Also, matched control's timing is subject to the timing of the mTBI group. It is unclear what "similar" timing is meant for healthy, matched controls. There are concerns about the specificity given for the inclusion/exclusion criteria, specifically about the type of TBI injury subjects had and clarity about the psychological disorder. Minor weaknesses of the study include that the external monitor has not been identified and there isn't a medical monitor or data and safety monitoring board.

Statistical Plan and Data Analysis
Average Score: 5.8

Scientist Reviewer A

Strengths: The statistical plan has some minor strengths. The applicants have included some specifics around the various regression models they anticipate using for Aim 1, and a mixed-effects model approach to address Aim 2. They appear to have given some consideration to confounds and interactions of interest.

Weaknesses: The application has some moderate weaknesses for the statistical plan. While they do include some specifics for the analytic approach for each aim, the analysis plan for each aim is generally a bit broad and somewhat generic in places. For example, the application includes examples for the various confounds and interactions, but then states "etc." for both, which suggests some uncertainty around how many models will be examined, which could in turn lead to the detection of spurious effects. The applicants plan to follow rigorous rules to guarantee reproducibility of their data analysis results but it is unclear what this means because the statement is quite generic and unspecific.

Power analyses are included for each aim. However, the details for each power analysis are limited, and it is not clear exactly what values were used to derive the power estimates. For example, for Aim 1, the applicants state that they will "investigate the minimum detectable effect size of mTBI history on mean heart rate difference between resting and exertion." The details used to derive the power values in Table 6 are quite unclear, based on this statement and generally short description. The power analysis for Aim 2 is equally unclear and rather broad and nonspecific.

Scientist Reviewer B

Strengths: There were power analyses performed for both aims to ensure that the proposed participant recruitment numbers are adequate to ensure the study is well powered. The proposed participant recruitment goals are feasible given the time line, and the proposed statistical analyses are appropriate and suitable to answer the research questions. In addition to the proposed analyses, the applicants have included alternative approaches, including how they will handle missing data in the analyses and what they will do if there is a convergence issue due to the small sample size in Aim 2. It is clear that the applicants have taken careful consideration of the analyses planned for this proposal.

Weaknesses: The power analysis for Aim 2 is insufficiently described with respect to the study design. The subanalysis of the 20 mTBI individuals is not entirely clear.

Biostatistician Reviewer

Strengths: Statistical methods that will be used are described accurately. Measures and outcomes that will be collected are clearly provided in 3 tables. These are major strengths.

Weaknesses: The following are noted as significant weaknesses. The statistical technique that was used for the power analysis for Aim 1 is not clear. Also, it is not clear if they aim to compare 20 service members with mTBI versus 20 college athletes with mTBI. It is unclear where n = 20 comes from and whether it is an assumption. The power analysis calculations do not appear to have used at least one of the regression methods that will be used for analyses. The power analysis (sample size justification) for Aim 2 is not appropriate because it seems mixed models were not used even though they say this method will be used to analyze the longitudinal data.

Data and Research Resources Sharing Plan
Average Score: 7.0

Scientist Reviewer A

Strengths: The application contains several major strengths for data and research resource sharing. The applicants have noted that data will be shared with the TBI research community through the Federal Interagency Traumatic Brain Injury Research (FITBIR) database and have stated that they will ensure that appropriate data will be conformed to common data elements (CDEs.) The applicants note that findings will be disseminated via peer-reviewed publications and presentations at scientific meetings. The applicants include appropriate discussion of which selected outcome measures are consistent with TBI CDEs to ensure sharing with FITBIR.

Weaknesses: The application includes some moderate weaknesses. The Resource Sharing section of the data sharing plan has limited description with little detail, essentially one rather generically worded sentence. Description and detail about what and how it might be shared with pertinent military communities are lacking, particularly given the focus of the proposed research on collaboration with USASOC. The applicants note that data from some potential participants may not be easily shared with FITBIR because of security clearance reasons. Given the sensitivities around collection and sharing of that data, one would expect a similar discussion and/or caveats around sharing of lessons learned or resources in the resource sharing section of the plan.

Scientist Reviewer B

Strengths: The applicants have provided a plan for sharing the findings from deidentified data of the study results in both peer-reviewed publications and at national scientific meetings. The applicants provide transparent examples of how the participants will be coded and deidentified to protect their privacy. The applicants have included that the data will be shared through FITBIR. There is an adequate data sharing plan in place for the proposed study, and the applicants have taken special considerations to ensure that the necessary security clearance measures will be in place for the data of combat service members. The applicants have budgeted for a data analyst to ensure the data conform to the necessary CDEs.

Weaknesses: While the discussion of special considerations of necessary security clearance measures is a strength, there is limited discussion of how this would be handled from a resource sharing standpoint. This is a minor weakness.

Transition Plan
Average Score: 7.2

Scientist Reviewer A

Strengths: The application's transition plan includes several major strengths. The applicants have noted that the outcomes of their 2 aims (characterizing physiological underpinnings of mTBI and their relationship to functional performance and recovery trajectory) will enhance clinical evaluations and return to duty protocols for service members after mTBI. They have identified a 3-pronged strategy to disseminate results, translate to clinical practice, and continue to the next phase of development via additional studies. This approach seems reasonable and logical. The applicants have described their strong relationships with both the DOD and industry partners and successful history of securing funding. These relationships are appropriate for the proposed plan. The applicants have specified a 5-year plan that identifies an approach for dissemination of findings to the scientific and military communities, larger data collections with a more heterogeneous population, and additional integration into the military environment. This plan generally seems achievable.

Weaknesses: Some minor weaknesses were noted. The intellectual property discussion is somewhat generic describing a general approach and interaction with the Office of Technology Commercialization if appropriate, and does not seem to specifically identify if or how the government will be able to access developed products or technologies that may arise from the proposed research. The risk analysis for the transition plan is sparse, with focus more on generic research issues (ie, confounds and statistical modeling) as opposed to specifics for the proposed plan.

Scientist Reviewer B

Strengths: The applicants propose 3 key strategies to share their study findings and translate them into clinical practice: (1) study publications, (2) clinical translation with the Department of Defense by altering existing clinical tools where study findings could be included to improve clinical practice, and (3) development of future trials based on the results of this study to improve military health in a larger sample. There is a clearly defined plan for the protection of intellectual property if the study findings result in technological advancements.

Weaknesses: There is limited discussion on the risk analysis of the proposed project. This is a minor weakness.

Technology Transfer Specialist Reviewer

Strengths: The transition plan has a number of moderate strengths. Plans for knowledge product dissemination are satisfactory. Plans for continuity of funding for future studies are plausible. The team's history and track record with extramural funding bode well for grant funding of future studies. Proposed time lines for dissemination of knowledge product are reasonable. The proposed transition plan strategy has negligible regulatory barriers to entry other than typical human subjects research considerations. The hardware technology being used is commercially available; this not a limitation to widespread adoption of the methodology.

Weaknesses: The strategy to push the assessment technology into the clinical community of practice is not highly developed. The transition plan doesn't sufficiently address whether the assessment tools being used (Modified Balance Error Scoring System, Military Acute Concussion Evaluation-2, Sport Concussion Assessment Tool 5th Edition, PCL-5) require permission of licenses but this is a minor criticism. The reliance on the technology transfer office for commercialization does not clearly specify proprietary strategies (patents, copyrights, software distribution strategies, content licensing strategies) for new assessment tools, again a minor criticism.

UNSCORED CRITERIA

Community-Based Participatory Research

Scientist Reviewer A

The community-based participatory research (CBPR) approach is well-defined and comprehensive. The application has letters of support that clearly describe roles and lived experience from active duty service members, civilians who work directly or indirectly with service members, and veterans. This diverse group will bring important perspectives and experiences to the proposed research. The applicants have noted that civilian CBPR participants will be encouraged to complete Green Zone training to learn more about military experiences, and that data analysis basics will be provided to all CBPR participants to ensure a common understanding for discussion and interpretation of results from the proposed study. The applicants have noted that an equitable decision-making approach will be used and the CBPR members will be involved in decision-making for many aspects of the proposed research (eg, interpretation of data, dissemination of findings). The applicants additionally note that they will leverage previously established research practices that include community partners as applicants on scientific manuscripts to facilitate the sharing of information that will ideally be translated into policy changes that impact the various partner/stakeholder communities.

Scientist Reviewer B

It is clear that this proposal is well supported by the applicants' community partners, both on the military and civilian sides. There are ample letters of support from their community advisory board, and the table of board members is a great addition to the organizational structure of the proposal. The study site has a well-documented history of successfully working with military partners, and this is clear from the application.

Budget

Scientist Reviewer A

The direct costs for the proposed research are within the amount allowed. The budget generally seems appropriate for the proposed research.

Scientist Reviewer B

The direct costs do not exceed the allowable direct costs. The budget justification is appropriate for the proposed research.

Environment

Scientist Reviewer A

The scientific environment and institutional resources are appropriate and adequate for successful execution of the proposed research. Letters of support from the various institutions are appropriate and compelling. Available study space and resources are adequate.

Scientist Reviewer B

The UNC-Matthew Gfeller Center Simulation, Training, Analytics, and Rehabilitation (STAR) Heel Performance Lab, and Department of Family Medicine are well-equipped to handle the needs of the proposed study. There are no weaknesses associated with the environment of the proposed study.

Application Presentation

Scientist Reviewer A

The application was well-formatted and easy to read. The writing was clear.

Scientist Reviewer B

This was a well-written proposal that was clear and easy to read. This project has a large number of outcome measures, and the inclusion of the tables throughout the research strategy was a strong addition that made the aims and outcomes easy to follow. A minor weakness was the extensive use of acronyms throughout the proposal. It was hard to keep track of all the acronyms at points throughout the document.
